# Computational Modeling of Collateral Circulation and Arteriolar Vasodilation in Ischemic Tissue Perfusion

**DOI:** 10.1007/s10439-026-04052-6

**Published:** 2026-03-13

**Authors:** Nolan Moreaux, Chang Min Lee, Bon-Kwon Koo, Keun-Hwa Jung, Jung-Kyu Han, Hyeyeon Chang, Hyun Jin Kim

**Affiliations:** 1 Department of Mechanical Engineering, Korea Advanced Institute of Science and Technology, Daejeon, Republic of Korea; 2 Department of Mechanical Engineering and design, Univ. Bourgogne Franche-Comte, UTBM, Sevenans, France; 3 Seoul National University Hospital, Seoul, Republic of Korea; 4 Konyang University Hospital, Nonsan, Republic of Korea

**Keywords:** Blood flow modeling, Collateral circulation, Arteriolar vasodilation, Perfusion simulation, Ischemia

## Abstract

**Purpose:**

Despite substantial advances in computational cardiovascular modeling, simplifying assumptions often overlook key compensatory mechanisms. To better capture physiological responses under pathological conditions, we incorporate two essential compensatory mechanisms—collateral circulation and arteriolar vasodilation—into a multiscale blood perfusion simulation framework.

**Methods:**

Collateral vessels are stochastically generated to supply ischemic regions, with model parameters systematically varied to control vessel density, caliber, and spatial distribution. Arteriolar vasodilation is modeled to represent the combined response of vascular smooth muscle cells to local metabolic and hemodynamic stimuli. To assess their impact on perfusion, artificial stenoses are introduced into both an idealized geometry and a subject-specific cerebrovascular model.

**Results:**

Simulation results across varying collateral configurations and vasodilation capacities demonstrate that perfusion enhancement from sub-resolution collateral vessels alone is limited and highly dependent on vessel caliber and density. In contrast, combining collateral flow with vasodilation produces a more pronounced improvement in tissue perfusion. These findings suggest that arteriolar vasodilation serves as the primary mechanism for ischemic compensation, with collateral circulation providing secondary support which is consistent with clinical observations.

**Conclusion:**

The proposed methods enable multiscale blood flow simulations while accounting for the main autoregulatory mechanisms. The models have been verified using literature data and are shown to provide accurate results.

## Introduction

Tissue perfusion is sustained by the vascular network that delivers blood to the tissue and is regulated to meet local metabolic demands via a combination of active and passive mechanisms. Key regulatory processes include modulation of vascular tone, systemic blood pressure control, heart rate modulation, and myogenic responses [13, 14, 47]. Additionally, in response to regional perfusion deficits, collateral vessels may form or undergo structural remodeling to restore adequate blood supply [17]. While central nervous system control plays a significant role in perfusion regulation [52], additional mechanisms—such as passive mechanical responses, collateral flow redistribution, and locally driven hemodynamic adjustments—also contribute to perfusion. Under pathological conditions, a range of autoregulatory and compensatory mechanisms are engaged to maintain homeostasis. These include both short-term functional responses and long-term vascular remodeling across multiple temporal scales, all aimed at preserving sufficient perfusion to tissues at risk.

Among the various regulatory mechanisms, two play a particularly significant role in maintaining perfusion under pathological conditions: the formation and recruitment of collateral networks and the vasodilation of arterioles downstream of the affected vessel [9, 18, 30, 43, 45]. Collateral circulation provides alternative pathways for blood flow, while arteriolar vasodilation reduces local vascular resistance, thereby enhancing flow through the primary arterial pathways. Both mechanisms act to decrease overall resistance and improve blood supply to the affected tissue. The critical roles of collateral vessels and arteriolar vasodilation in regulating perfusion have been demonstrated in numerous experimental and computational studies. However, these investigations are often limited by the difficulty of directly measuring microvascular parameters, which are typically below the resolution of conventional imaging modalities. As a result, the individual contributions of these mechanisms are frequently inferred through indirect measurements. Moreover, the relative effectiveness of vasodilation and collateral recruitment can vary depending on disease location, severity, and physiological context, suggesting that the dominant mechanism for restoring perfusion may differ across clinical scenarios.

Numerous experimental studies have investigated the effects of vasodilation [22, 27, 32, 51] and collateral circulation [4, 5, 8, 16, 19, 21, 23, 31, 42, 48] on tissue perfusion [49]. Boer et al. [11] demonstrated a negative correlation between collateral vessel density and the volume of infarcted brain tissue, highlighting the importance of collateral pathways in maintaining perfusion. Similarly, Václav$\mathring{u}$ et al. [50] reported a strong association between vasodilatory capacity and cerebral blood flow reserve in patients with cerebrovascular disease; however, their analysis did not account for the influence of collateral circulation. In practice, isolating the individual contributions of collateral flow and vasodilation remains challenging due to the physiological coupling of these mechanisms and the fact that most clinical perfusion measurements reflect their combined effects.

Computational methods have been increasingly employed to investigate the role of arteriolar vasodilation and collateral circulation in regulating blood flow under pathological conditions. For modeling vasodilation, Hudetz et al. [25] developed a one-dimensional cerebrovascular model incorporating vasodilatory mechanisms to evaluate blood flow alterations associated with cerebrovascular disease. Boileau et al. [12] implemented a vasodilation model for coronary artery disease to assess disease severity via flow-based metrics. Moorhead et al. [36] introduced a compensatory, vasodilation model to restore blood flow downstream of a stenosis. Rundfeldt et al. [41] proposed a multiscale model integrating synthetic arteriolar vasodilation to estimate the hemodynamic impact on cerebrovascular disease in patient-specific simulations.

For modeling collateral circulation, many groups including Blanco et al., Alastruey et al., and Spaan et al. [6, 10, 46], implemented a simplified zero-dimensional collateral network model to examine the influence of collateral pathways in a stenosed vascular model using an idealized geometry. Liang et al. and Anbazhakan et al. [7, 34] extended this concept by introducing a one-dimensional collateral network to evaluate stenosis severity based on collateral flow contributions. Otani et al. [38] developed a multiscale blood flow simulation framework incorporating collateral pathways to quantify their role in tissue perfusion. Similarly, Padmos et al. [39] constructed a surface-level collateral network of the brain to investigate its influence on cerebral blood flow distribution.

Numerous computational studies have investigated the individual effects of arteriolar vasodilation and collateral circulation on blood flow regulation. However, the combined impact of these two key autoregulatory mechanisms and their relative contributions to perfusion compensation have not been systematically explored. In experimental settings, isolating and quantifying the effects of each mechanism is challenging due to their strong physiological coupling and the limitations of current measurement techniques. Computational modeling, on the other hand, enables the independent analysis of vasodilatory and collateral responses and allows for systematic evaluation under controlled conditions. Furthermore, computational approaches facilitate the incorporation of parameter uncertainties, thereby capturing the inter-subject variability observed in clinical populations and enhancing the generalizability of the findings.

In this study, we developed a multiscale blood flow simulation framework that incorporates both collateral circulation and arteriolar vasodilation. Uncertainties in collateral network morphology and vasodilatory capacity were systematically accounted for using both idealized and subject-specific vascular geometries. The model enables quantitative assessment of the relative contributions of these two compensatory mechanisms to tissue perfusion within ischemic regions. This approach provides insight into their respective roles in blood reperfusion and allows for a comparison of the energetic and structural requirements for collateral vessel development versus the functional capacity of these vessels to restore flow. Finally, we conducted simulations across a range of collateral configurations and vasodilation capacities under varying degrees of arterial stenosis in a subject-specific cerebrovascular model to evaluate their combined and individual effects on perfusion.

## Materials and Methods

**Fig. 1:**
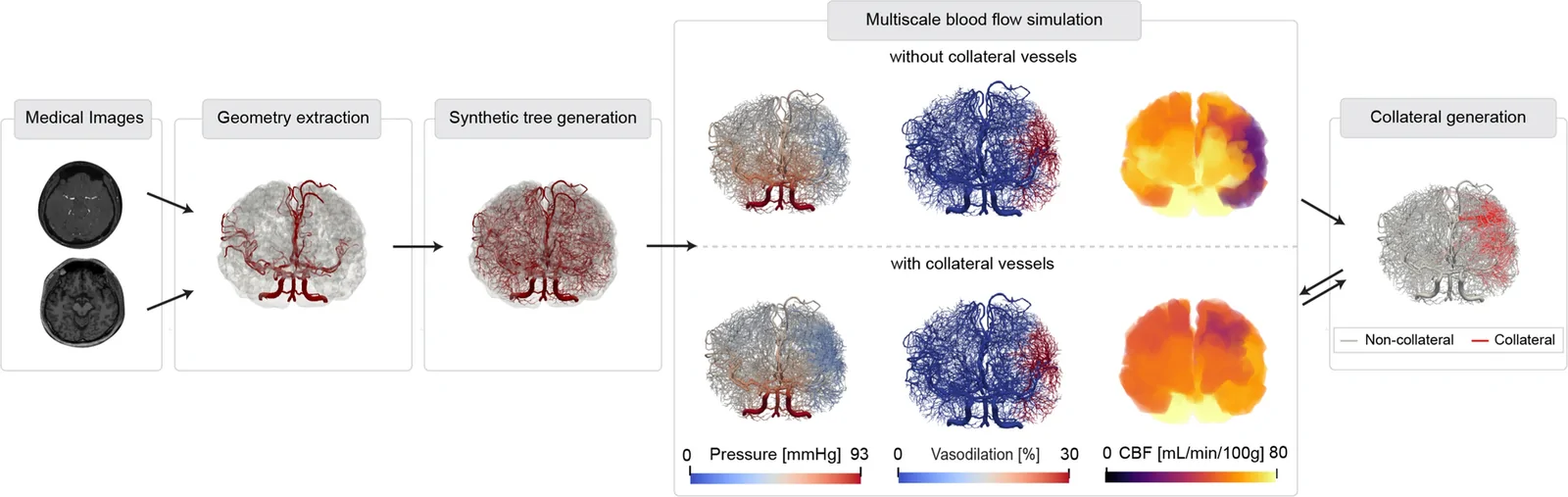
Workflow of the multiscale blood flow simulation, including collateral vessel generation and arteriolar vasodilation modeling.

**Fig. 2:**
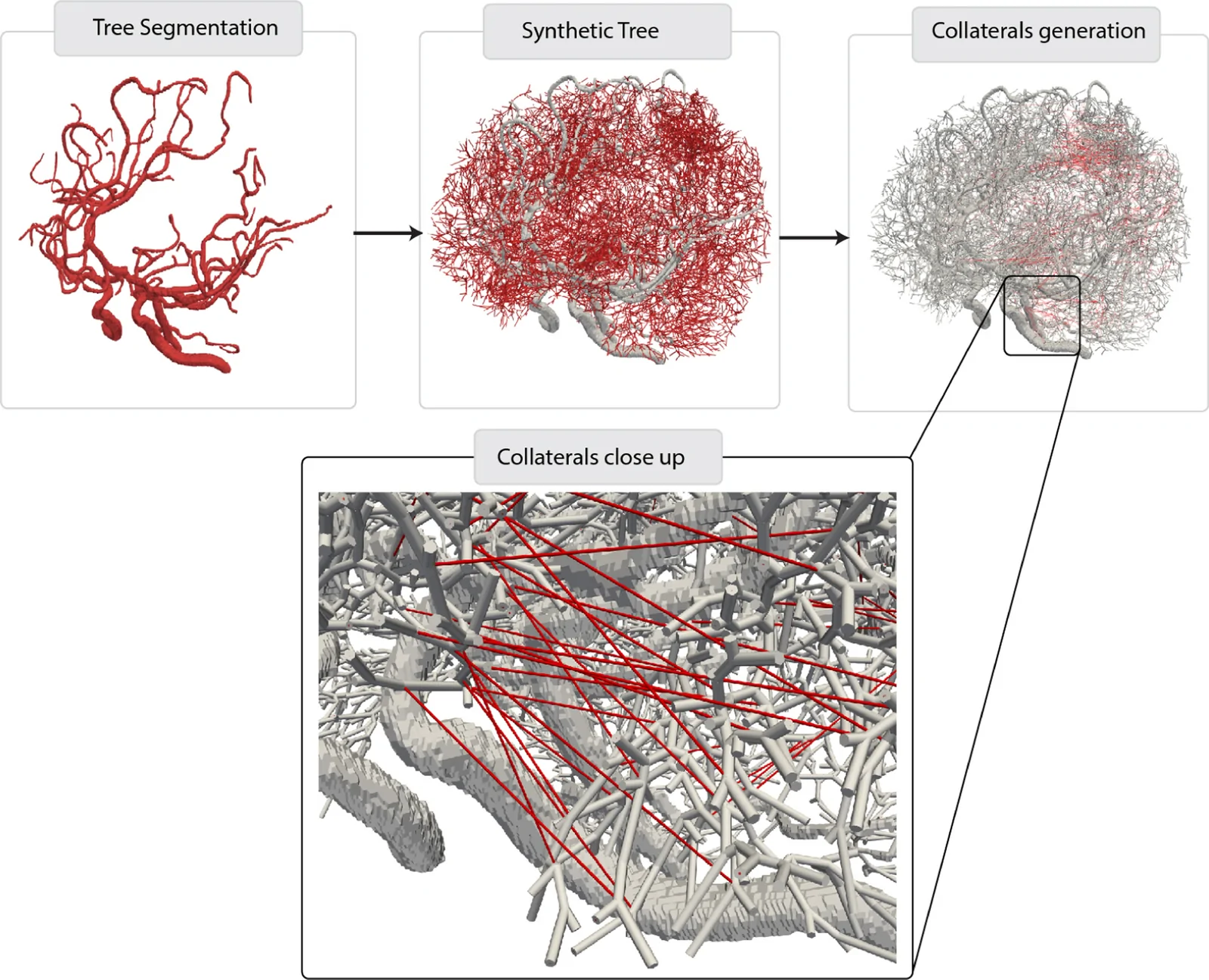
Tree generation steps. Top: Tree segmentation from medical imaging. Middle: synthetic tree generation (red) from segmented tree. Bottom: collateral generation (red) from synthetic tree.

The overall workflow for collateral construction and perfusion simulation is illustrated in Fig. 1. Closer details for geometry construction are seen in Fig. 2. The process begins with medical imaging data from which vascular structures and corresponding tissue regions are extracted. To simulate perfusion beyond the resolution limits of the imaging data, a growth-based vascular tree generation algorithm is employed [29]. This algorithm extends the arterial network by synthesizing sub-resolution vessels based on supply–demand relationships and the minimum work principle [37]. Once the extended vascular network is established, blood flow and pressure are computed to estimate tissue perfusion. Collateral vessels are then introduced into the model, followed by repeated flow simulations incorporating arteriolar vasodilation. The final steps—collateral generation and multiscale perfusion simulation with arteriolar vasodilation if necessary—can be iteratively applied to explore various physiological scenarios and assess model sensitivity.

The Material and Methods section provides a detailed description of the key components of the simulation framework and is organized into four subsections. The first subsection outlines the construction of the imaged-based vascular structures and tissue regions and subsequent vascular tree generation. The second subsection presents the governing equations used to compute blood flow and pressure within the multiscale vascular network and associated perfused tissue. It also describes arteriolar vasodilation integrated with the governing equations. The third subsection outlines the computational strategies developed to generate collateral vessels. The fourth subsection describes the anatomical geometries and simulation details presented in the Results section.

### Geometry Construction

#### Construction of Vascular Structures and Tissue Regions

Vascular structures and tissue regions are extracted from medical imaging. For this purpose, a 3T MRI was used, leading to a spatial resolution of 0.25mm*0.25mm*1 mm. The data are then imported 3D Slicer [28] for arteries segmentation. Here, a threshold method is used to keep only the arteries before separating merged arteries and remove artifacts generated by noise. Then, centerline and vessel radius are determined using Voronoi Tesselation. As for the tissue geometry, FreeSurfer [20] is used to extract different part of the brain and separate them. From these groups, white matter, gray matter, thalamus, caudate, putamen, and pallidum are kept for simulation. The model is finally meshed and ready for simulation.

#### Generation of Synthetic Trees

We generate synthetic vascular trees using a growth-based algorithm that links vessel development to the expansion of the tissue they perfuse. The final network combines two parts: an image-derived initial tree, described in previous section 2.1.1, and synthetic branches that represent vessels below imaging resolution. All terminal points of the segmented tree are treated as potential sources. Those located within or near the tissue domain are kept and assigned the minimum possible radius. Their initial flow supply is set according to standard physiological rules. Using these flow estimations, we define the initially perfused tissue region based on known relations between supplied flow and viable tissue volume. At each iteration, every terminal vessel supplies a specific tissue region computed through a direction-aware partitioning method. The tissue then expands from the current domain to a larger one, representing metabolic growth. New vessels are added whenever the enlarged domain requires additional flow. The number of new terminal branches directly reflects the increase in tissue demand. Each new terminal branch is attached to the existing network at a location that minimizes a physiological cost balancing viscous efficiency and metabolic maintenance. Vessel radii and segment geometry adapt automatically to the local tissue demand and the structure of the surrounding tree, ensuring consistent supply across scales. Growth proceeds until the synthetic tissue domain matches the target tissue volume derived from imaging. The final vascular tree delivers the required physiological perfusion while preserving realistic topology, geometry, and hemodynamic behavior.

### Governing Blood Flow Equations

The governing equations for blood flow comprise two primary components: a one-dimensional (1D) flow model applied to the explicitly represented vascular network which includes arteries, arterioles, and collateral vessels, as depicted in Fig. 1 and a Darcy flow model [29, 35] used to represent capillary perfusion within the tissue, which is treated as a porous medium. These two systems are explicitly coupled: pressures at the terminal segments of the 1D vascular network serve as boundary conditions for the Darcy model, while the resulting tissue perfusion flows are fed back into the 1D equations. The two solvers are solved iteratively to ensure convergence and consistency between the vascular and tissue domains by explicitly coupling tissue flows and terminal blood pressure values.

#### One-dimensional Blood Flow Equations

Blood flow within the generated vascular networks is modeled using a one-dimensional (1D) approximation of the Navier–Stokes equations. In this study, vessel wall deformability is neglected, and the vessels are assumed to be rigid. The governing equations consist of the conservation of mass and the balance of linear momentum, solved along the axial direction *z* for each vessel segment as follows:

Mass conservation:

1 $$\begin{aligned} \frac{\partial Q}{\partial z} = 0 \end{aligned}$$

Momentum balance:

2 $$\begin{aligned} \frac{\partial Q}{\partial t} + \frac{\partial }{\partial z}\left[ \frac{Q^2}{S}\left( 1+\delta \right) \right] + \frac{\partial p}{\partial z}\frac{ S}{\rho } = S \cdot f + N \frac{Q}{S}, \end{aligned}$$

where *Q* denotes the volumetric flow rate, *p* the pressure, and *S* the cross-sectional area of the vessel. The parameter δ is the velocity profile correction factor, ρ is the fluid density, and $N = 8 \pi \nu$ represents the viscous loss coefficient for a parabolic velocity profile [26, 29], where ν is the kinematic viscosity of blood. Blood is approximated as a Newtonian incompressible fluid, and *f* denotes external forces per unit mass acting on the fluid. This 1D formulation is appropriate for explicitly modeled arterial and arteriolar conduits, where the flow occurs in actual vascular geometries rather than in a porous medium. The governing equations for all vessel segments are assembled into a global system and solved using a sparse LU decomposition algorithm [29].

#### Perfusion Simulation

In the tissue domain, blood flow is modeled using Darcy’s law for viscous flow through a porous medium, combined with mass conservation [29, 35]. The governing equations are expressed as follows:

3 $$\begin{aligned} & \vec {v} = - \frac{\kappa }{\mu }\nabla p \end{aligned}$$

4 $$\begin{aligned} & \nabla \cdot \vec {v} = \beta _{source}(p_{source} - p) - \beta _{sink}(p - p_{sink}), \end{aligned}$$

where $\vec {v}$ is the Darcy velocity, κ is the tissue permeability, μ is the dynamic viscosity of blood, and *p* is the local tissue pressure. The parameters $p_{source}$ and $p_{sink}$ represent the pressures at the arterial inflow (terminal segments of the 1D vascular network calculated with Eq. 2 and 3) and venous outflow boundaries, respectively. The coefficients $\beta _{source}$ and $\beta _{sink}$ are pressure-coupling parameters that characterize the hydraulic conductance of flow entering and exiting the tissue domain. The equations are discretized using a stabilized finite element method and solved with a generalized minimal residual (GMRES) solver, employing a block-Jacobi preconditioner [29, 35].

#### Boundary Conditions and Arteriolar Vasodilation

Two types of outlet boundary conditions were employed to solve the one-dimensional blood flow equations within the constructed vascular networks. At the arterial inlets, a mean aortic pressure of 93.3 mmHg was prescribed. At the outlets, flow boundary conditions were initially assigned based on the volume of the mapped tissue element supplied by each terminal vessel and were updated through feedback from the Darcy flow equations during the multiscale perfusion simulation. For each outlet-tissue pair, the target flow rate was computed as

5 $$\begin{aligned} q_{\text {outlet}} = \rho _{\text {tissue}} V_{\text {outlet}} m_{\text {tissue}}, \end{aligned}$$

where $V_{outlet}$ is the tissue element volume, $m_{tissue}$ is the baseline perfusion demand per unit mass, and $\rho _{tissue}$ is the tissue density. During the iterative perfusion simulation, $m_{tissue}$ was updated using the perfusion values returned by the three-dimensional Darcy flow solver.

Following this baseline simulation, outlets supplying hypoperfused tissue were subjected to arteriolar dilation using the vasodilation algorithm. For these outlets, the boundary condition was transitioned from a prescribed flow to a resistance boundary condition. This substitution is necessary because, once dilation begins, the outlet flow becomes an unknown variable governed by the one-dimensional flow equations. The outlet resistance was updated according to

6 $$\begin{aligned} R_{\text {outlet}} = \frac{R_{\text {outlet},0}}{d^{4}}, \qquad R_{\text {outlet},0} = \frac{p_{\text {ideal}}}{q_{\text {outlet}}}, \end{aligned}$$

with the constraint $\frac{R_{outlet,0}}{R_{outlet}} \in [d^{4}_{min}, d^{4}_{max}]$. Here, *d* is the vessel dilation ratio applied to the arteriolar segments, $d_{max}$ and $d_{min}$ are the maximal and minimal allowable dilation ratios, $q_{outlet}$ is the target flow rate defined in Eq. 6, and $p_{ideal}$ is the outlet pressure predicted by an ideal model. An ideal model is generated by temporarily removing any stenosis present in the vascular geometry to obtain an ideal estimate of downstream resistance without the confounding influence of disease or vasodilation.

Arteriolar vasodilation was applied to vessels within the initial diameter range of 6-100 μm [30] that belong to the non-collateral (primary) network. These vessels were permitted to dilate or constrict within predefined physiological limits. The dilation ratio *d* at each iteration was determined using

7 $$\begin{aligned} d = f (\frac{q_{\text {computed}}}{q_{\text {outlet}}}), \end{aligned}$$

where $q_{tissue}$ is the perfusion supplied to the corresponding tissue element and $q_{outlet}$ is the target flow rate defined in Eq. 6. This arteriolar vasodilation continues until either the allowable dilation capacity limit of each arteriole segment is reached or the target perfusion is achieved locally.

For all simulations assessing variations in collateral density and radius ratio, the same set of arteriolar vessels was used for vasodilation across cases to ensure consistency and enable meaningful comparison. The maximum allowable vasodilation and vasoconstriction ratios were treated as user-defined input parameters.

### Collateral Generation and Quantification

#### Collateral Generation

Collateral vessels were generated according to the following procedure. After constructing the synthetic arterial tree, a one-to-one mapping between terminal vessel segments and tissue elements was established. A multiscale blood flow simulation without collaterals and without arteriolar vasodilation was then performed to determine the perfusion status of each tissue element. Terminal segments supplying tissue with flow below the hypoperfusion threshold were labeled as diseased, and their upstream segments were recursively labeled as diseased unless at least one child segment was classified as healthy. All remaining segments were labeled as healthy. Collateral vessels were subsequently generated stochastically from healthy to diseased segments based on three input parameters: the hypoperfusion threshold, collateral density, and collateral radius ratio.

The hypoperfusion threshold specifies the minimum blood flow per unit tissue mass required to avoid infarction. In this study, a fixed value equal to 60% of the baseline perfusion was used. The collateral density defines the fraction of diseased vessel segments that receive a collateral connection. A density of 1 indicates that all diseased segments receive collaterals, whereas a density of 0.5 indicates that only half do. The radius ratio determines the radius of each collateral as a fraction of its donor vessel’s radius and ranges from 0 to 1.

Collateral creation proceeds iteratively as follows. A diseased segment is randomly selected, followed by a healthy donor segment chosen from physiologically plausible candidates. The radius of the collateral is assigned according to the prescribed radius ratio. A new collateral segment is then added between the midpoints of the donor and diseased segments. This process is repeated until the target collateral density is reached.

To ensure physiological plausibility, three guiding hypotheses were incorporated into the selection of donor and receiver segments. First, collaterals are assumed to have at least one end within the hypoperfused regions. While collaterals may also form with both ends in adequately perfused tissue, such cases were excluded because they are expected to contribute minimally to restoring flow in ischemic areas [33]. Second, collaterals are assumed to preferentially connect distinct arterial branches rather than forming within the same branch or between a vessel and its ancestor. To enforce this, a connectivity check ensures that the two candidate segments are not ancestor–descendant pairs, allowing connections between vessels sharing a common root but belonging to different sub-branches. Third, anatomical realism is maintained by constraining the radius of the donor and receiving vessels to be similar with 10% margin and below the minimum segmented radius in the imaging data, reflecting the assumption that larger vessels would otherwise have been visible during segmentation.

#### Collateral Quantification

To assess the hemodynamic feasibility and energetic cost of the constructed collateral networks, both metabolic and viscous energy expenditures were quantified. Metabolic energy expenditure was estimated by computing the total volume of all collateral vessels, defined as

8 $$\begin{aligned} \sum _{n} V_{n} \end{aligned},$$

where $V_n$ denotes the volume of the *n*-th collateral segment.

Viscous energy loss associated with collateral flow was computed as

9 $$\begin{aligned} \sum _{n} Q_{n} \Delta P_{n} \end{aligned},$$

where $Q_n$ is the flow rate through the *n*-th collateral and $\Delta P_n$ is the pressure difference between its proximal and distal ends.

These quantities were used to evaluate the energetic burden of the collateral network and to infer energetically favorable configurations based on the physiological principle of minimum work.

### Simulation Details

This study employs two distinct geometrical models for simulation. The first is an idealized spherical tissue domain with a radius of 5 cm. Two primary arteries, each with a radius of 0.5 cm, are artificially constructed to supply blood to the sphere. A 90% diameter stenosis is introduced in one of the arteries to simulate pathological conditions. To support multiscale perfusion simulations, smaller arteries and arterioles are synthetically generated within the domain. Two sets of simulations are performed using this geometry. In the first set, perfusion simulations are conducted while systematically varying collateral density and radius ratio. These simulations aim to quantify individual and combined contributions of collateral circulation to tissue perfusion. A normal tissue perfusion rate of 100 ml/min/100 g—representative of left ventricular perfusion—is assumed, and a hypoperfusion threshold is defined as 60 ml/min/100 g.

In the second set, collateral density and radius ratio are adjusted to approximate perfusion volumes corresponding to poor, moderate, and good collateral circulation, as categorized by Boer et al. [11]. Although Boer’s measurements were conducted in brain tissue, the spherical geometry is used here as a surrogate to explore the relationship between collateral density level and tissue perfusion. Target perfusion values and hypoperfusion thresholds specific to brain tissue were approximated based on reported physiological ranges. Normal cerebral blood flow (CBF) is usually between 50 mL/min/100 g and 80 mL/min/100 g [18, 30, 45]. In this study, we assume a uniform CBF of 65 mL/min/100 g. A series of simulations with varying collateral densities and radius ratios were conducted in a trial-and-error manner to identify configurations that closely match Boer et al.’s reported perfusion volumes. For each collateral density level—poor, moderate, and good—five simulations with different randomized collateral configurations are performed to evaluate the variability in perfusion outcomes due to stochastic collateral vessel generation. Subsequently, simulations were performed across varying collateral densities, radius ratios, and arteriolar vasodilation capacities to evaluate the relative contributions of collateral circulation and vasodilation.

The second geometry consists of a subject-specific brain tissue model coupled with a corresponding cerebrovascular network. This model was reconstructed from magnetic resonance imaging (MRI) data acquired under Institutional Review Board (IRB) approval of Seoul National University Hospital (IRB No. 2305-145-1434) and represents a 48-year-old male patient who experienced a transient ischemic attack associated with stenosis in the left middle cerebral artery (LMCA). Medical image data are acquired via various imaging modalities from which vascular structures and corresponding tissue regions are extracted using various imaging segmentation and post-processing tools such as U-Net [40], thresholding, SimVascular [2], 3DSlicer [28], VMTK [3], MeshMixer [1], and FreeSurfer [20], respectively. Due to insufficient imaging resolution to precisely quantify the degree of stenosis, artificial diameter narrowings of 60 %, 70 %, and 80 % were introduced to simulate varying severities. Major cerebral arteries were segmented directly from the imaging data, while smaller arteries and arterioles were synthetically generated to support multiscale perfusion simulations [29]. Simulations were performed under various combinations of collateral density, collateral radius ratio, and arteriolar vasodilation capacity to evaluate the individual and combined effects of these compensatory mechanisms on cerebral perfusion.

For brain tissue, a normal perfusion rate of 65 ml/min/100 g was assumed, and a hypoperfusion threshold of 39 ml/min/100 g was applied to identify regions at risk of ischemia. Parameter values for collateral density level—categorized as poor, moderate, and good—were adopted from those derived by conducting a sensitivity analysis on collateral sensitivity, radius, and hypoperfusion threshold using the idealized spherical geometry to ensure consistency across models.

The idealized spherical model was generated and simulated on a desktop equipped with an Intel Core i7-14700K (28 threads, up to 5.6 GHz) and 32 GB RAM (5600 MHz). The subject-specific cerebrovascular model was processed on a workstation equipped with an AMD Ryzen Threadripper PRO 5975WX (64 threads, up to 4.5 GHz) and 512 GB RAM (3200 MHz).

## Results

### Effect of Collaterals on Blood Perfusion Using Ideal Geometry

**Fig. 3:**
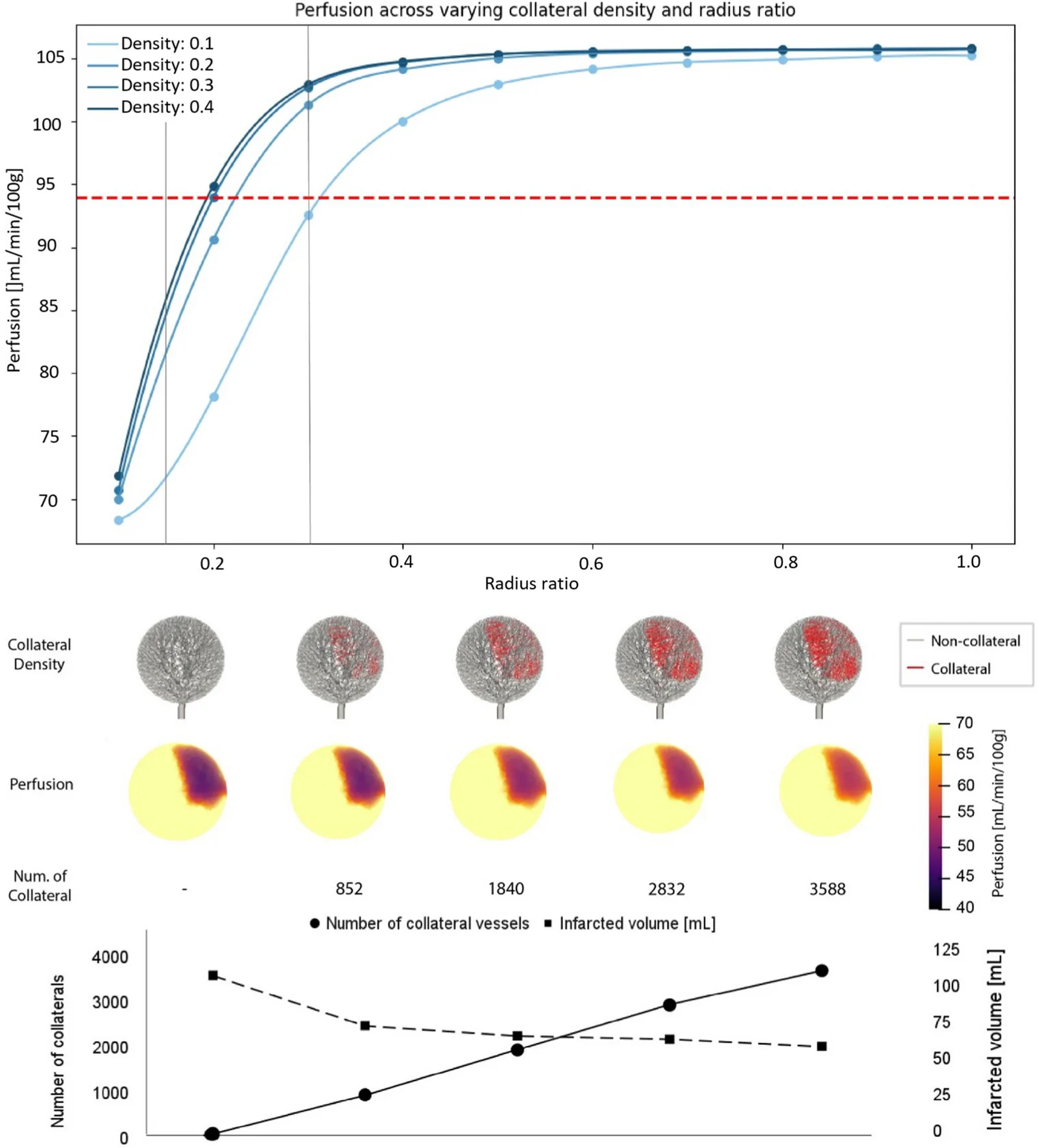
Top row: tissue blood flow across varying collateral densities and collateral radius ratios. Middle and bottom rows: perfusion simulation results for different collateral densities at a fixed radius ratio of 0.2 (indicated by the gray line in the top panel). The middle row shows collateral vessel configurations (red) for five density levels (absent, 10 %, 20 %, 30 %, and 40 %) with corresponding perfusion results. The bottom row displays the number of generated collateral vessels and the resulting infarct tissue volume for each case.

Synthetic vascular trees were generated for an idealized spherical tissue model with a radius of 5 cm. The resulting network comprised 49,464 vessel segments, with radii ranging from 8 μm to 1 cm, encompassing the full range from pre-capillary vessels to large arteries. A 90% diameter stenosis was artificially introduced in a vessel with a radius of 5 mm to simulate a pathological condition. A sensitivity analysis has been performed by using collateral radius and density of 0.15 and 0.3, respectively. Three tree resolution were generated (4939, 11858, and 49464 vessels) across all these configurations a maximum 2.6% change was observed. Therefore, the finest configuration has been chosen for better tissue perfusion simulation. Collateral vessels were generated using density values of 0.1, 0.2, 0.3, and 0.4, and radius ratio values ranging from 0.1 to 1.0 in increments of 0.1. A physiological tissue perfusion rate of 100 ml/min/100 g was assumed for healthy tissue. For each combination of collateral density and radius ratio, a collateral network was randomly constructed, and the resulting tissue perfusion was computed. The middle and bottom rows of Fig. 5 show the collateral absolute radius and length distributions for a density of 0.3. The top row of Fig. 3 illustrates the resulting tissue perfusion across the parameter space. The results indicate that larger collateral radius ratios can restore adequate perfusion regardless of collateral density, whereas smaller collaterals require higher densities to achieve sufficient tissue perfusion.

The middle row of Fig. 3 shows one computation for each collateral density for a radius ratio of 0.2. Vasodilation was excluded from these simulations to evaluate the contribution of collateral circulation alone.

**Fig. 4:**
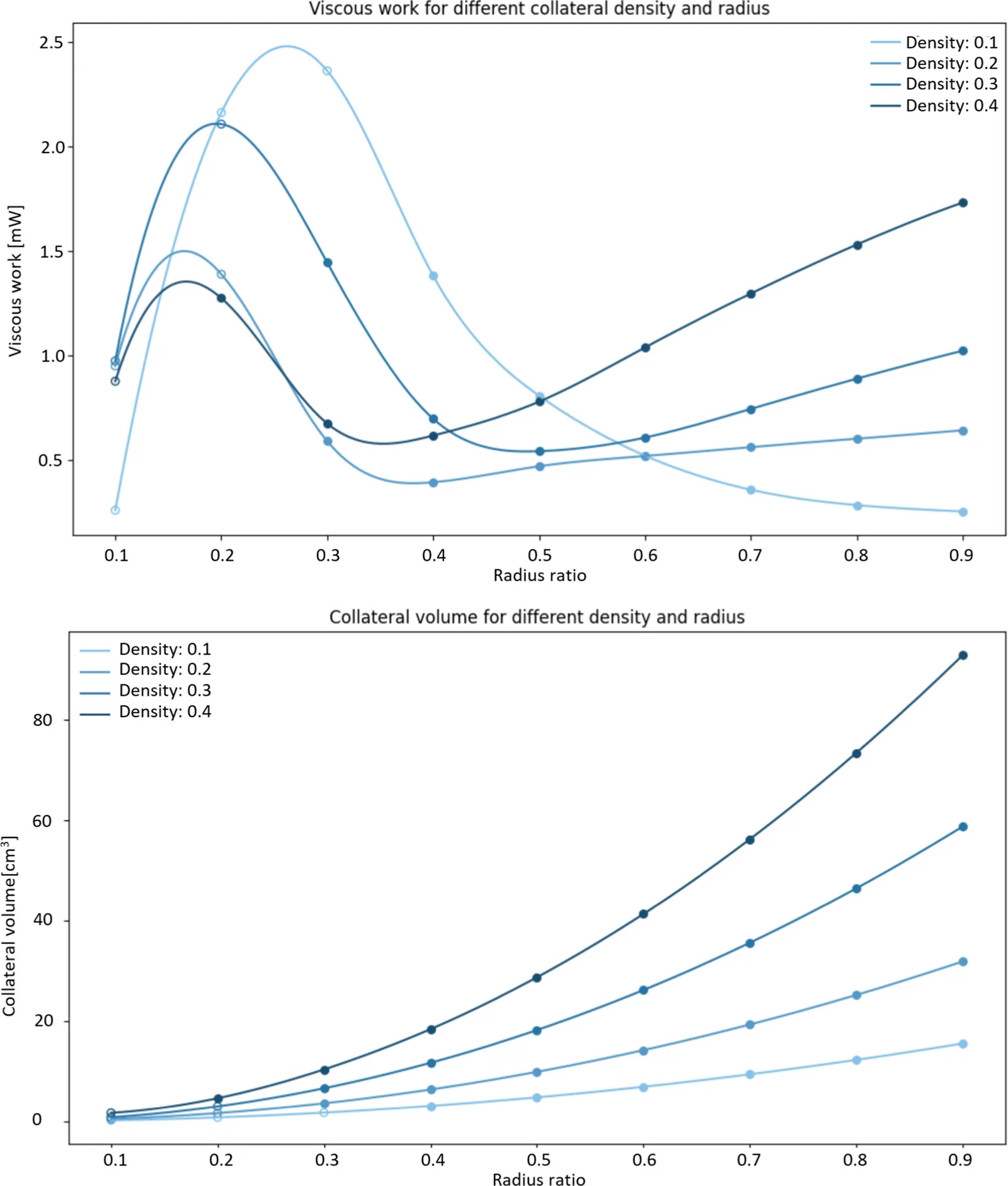
Top row: computed viscous energy loss as a function of collateral radius ratio for four collateral density levels. Bottom row: computed collateral volume as a function of collateral radius ratio for the same four density levels.

**Fig. 5:**
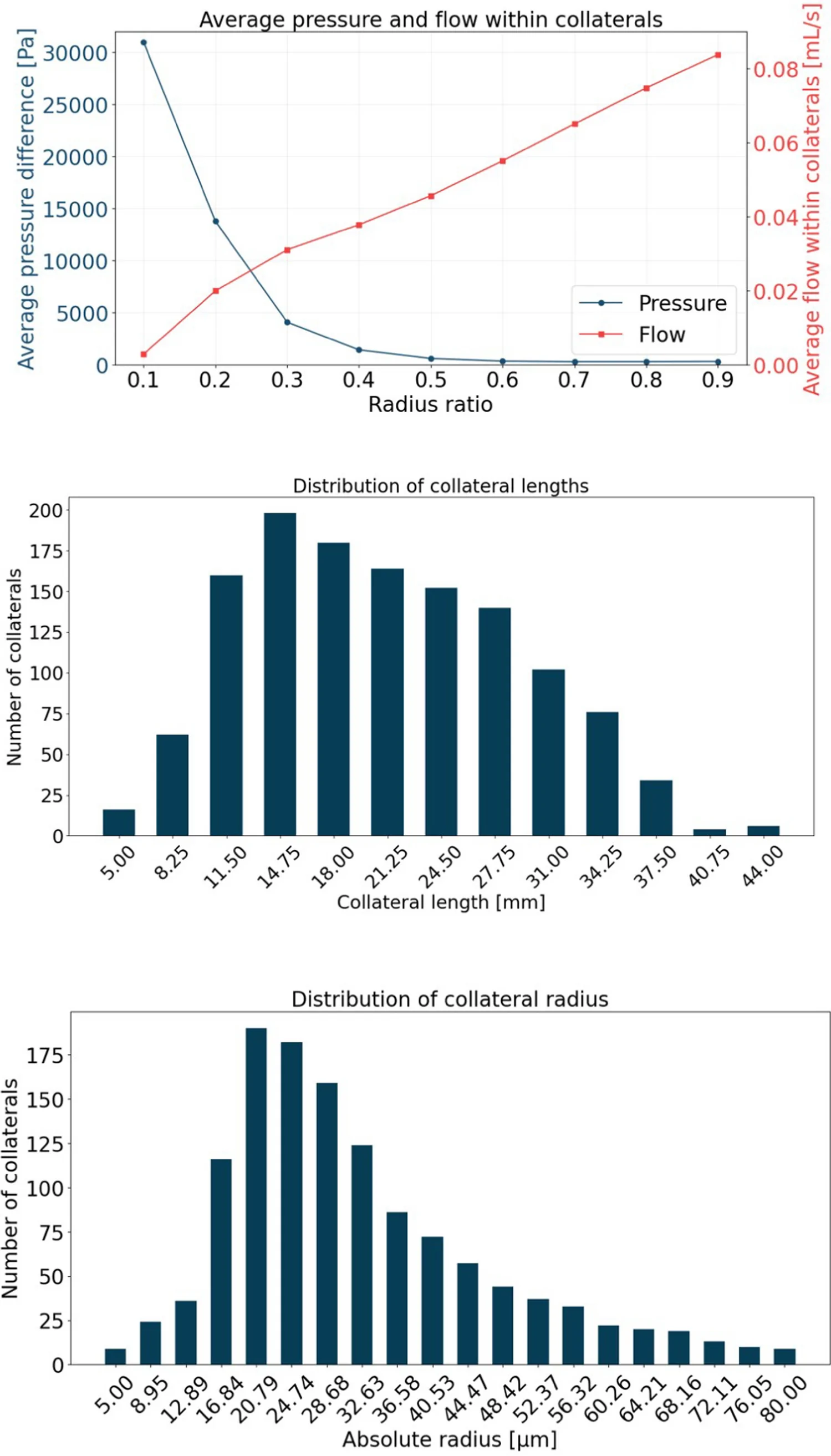
Top: Average pressure difference between both end of collaterals (left) and average flow within collaterals (right) accros varying radius ratio for a 0.3 density. Middle: Distribution of collateral by length for a density and radius ratio of 0.3. Bottom: Distribution of collaterals by radius for a density and radius ratio of 0.3.

Additionally, the metabolic and viscous energy loss associated with the constructed collateral networks were quantified, as shown in Fig. 4. The Pressure difference and collateral flow used to compute the viscous energy loss for the 0.3 density (details in section 2.3.2) can be seen Fig. 5. The total collateral vessel volume, which serves as a proxy for the metabolic work required to develop and sustain the network, increases monotonically with both collateral density and radius ratio values. In contrast, viscous energy loss exhibited a non-monotonic relationship with collateral radius.

### Effect of Collaterals and Vasodilation on Blood Perfusion Using Ideal Geometry

**Fig. 6:**
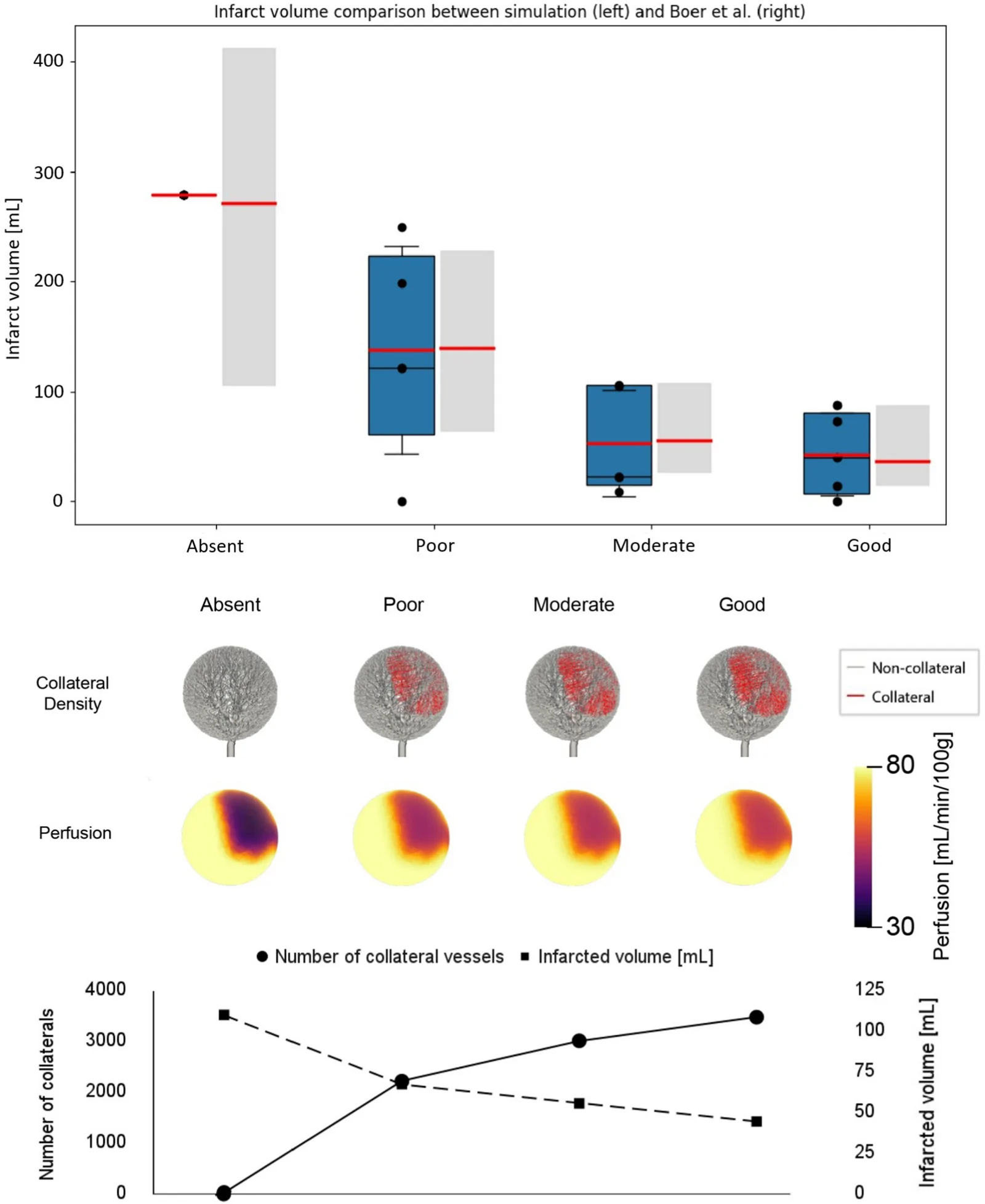
Top row: infarct volume [mL] across collateral density levels. Simulated values are shown on the left, reference values on the right. The red line indicates the mesensian, boxes represent the interquartile range (first-third quartiles), and whiskers denote the standard deviation. Middle row: collateral vessel configurations (red) for four density levels (absent, poor, moderate, good) with corresponding tissue perfusion results. Bottom row: number of generated collateral vessels and resulting infarct tissue volume for each case.

The same ideal geometry was utilized to replicate blood perfusion levels reported in the literature and evaluate the impact of collateral vessels and vasodilation on perfusion. Based on prior simulation results, a fixed collateral radius ratio of 0.15 was adopted. The corresponding collateral density values for poor, moderate, and good collateral grades were determined to be 0.245, 0.345, and 0.365, respectively. These radius/density combinations have been chosen based on the reperfusion values and homogeneity. Indeed, increasing radius would increase local perfusion, but the density will therefore decrease so much that the reperfusion would not be homogeneous within the infarct volume, due to the scarcity of collaterals. Moreover, as shown on top row of Fig. 3, choosing a collateral radius ratio of 0.3, thought to be more efficient on a viscous loss point of view, only lead to a perfusion increase of 20%, which remains insufficient to restore perfusion to non-ischemic levels. For each parameter set, collateral networks were stochastically generated, and tissue perfusion variability was evaluated through five independent simulations. The resulting standard deviations in perfusion were 84.4, 43.2, and 33.6 for poor, moderate, and good collateral densities, respectively. Note that the simulated configuration without collaterals have no standard deviation due to the randomness being associated with collateral generation only. Figure 6 presents the distribution of tissue perfusion across different collateral densities, along with comparison to perfusion measurements reported by Boer et al. [11] and computation results for each collateral density.

**Fig. 7:**
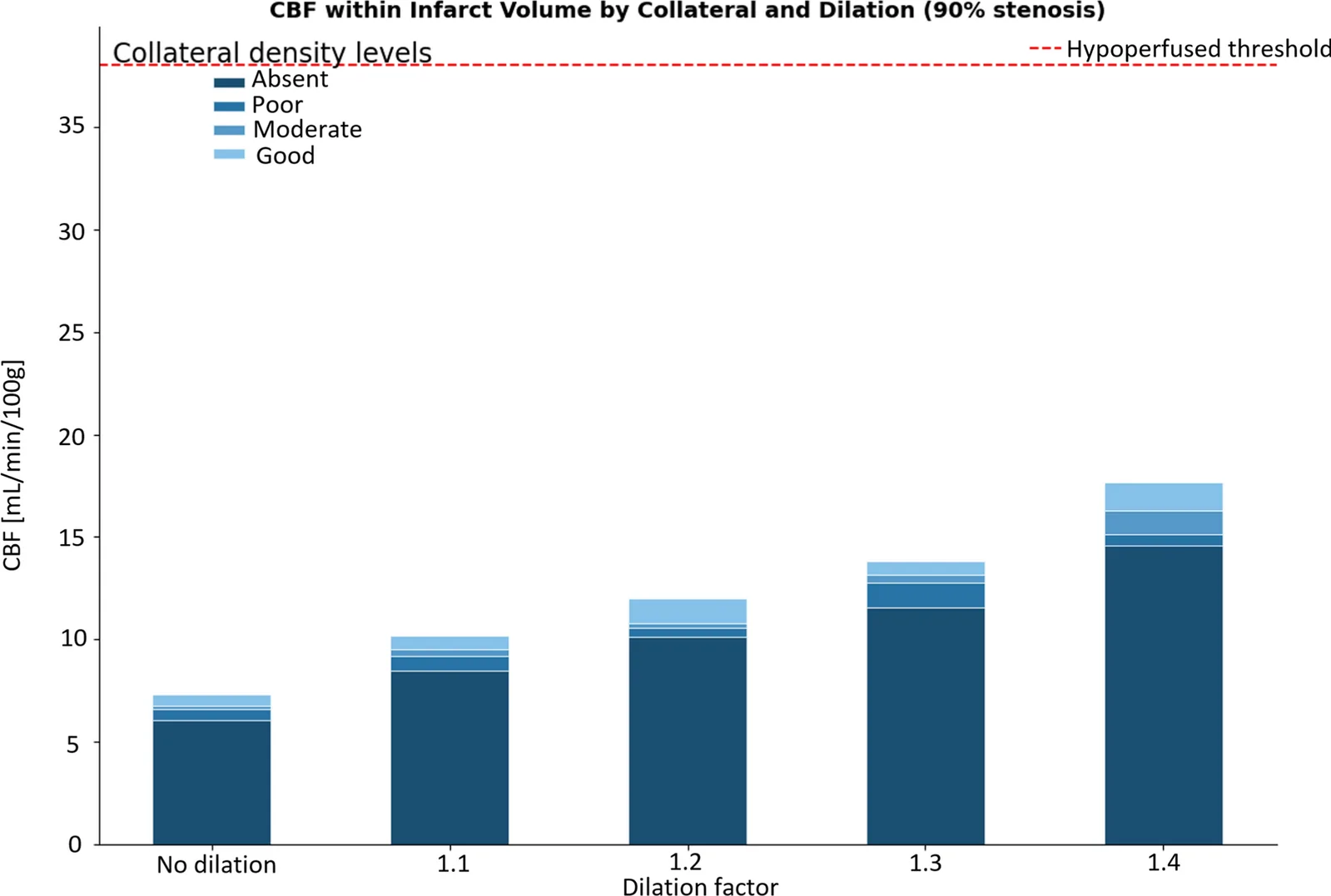
Mean cerebral blood flow [mL/min/100 g] within the initial infarct volume for four collateral density levels and five vasodilation levels under 90% stenosis for an idealized model.

Finally, the impact of vasodilation and collaterals was assessed by conducting simulation for different levels of vasodilation capacity (0%, 10%, 20%, 30%, and 40%). For each vasodilation level, four collateral density levels were simulated (absent, poor, moderate, and good). The results for all collateral density and vasodilation levels are presented in Fig. 7.

### Effect of Collaterals and Vasodilation on Blood Perfusion for Subject-Specific Cerebrovascular Model

**Fig. 8:**
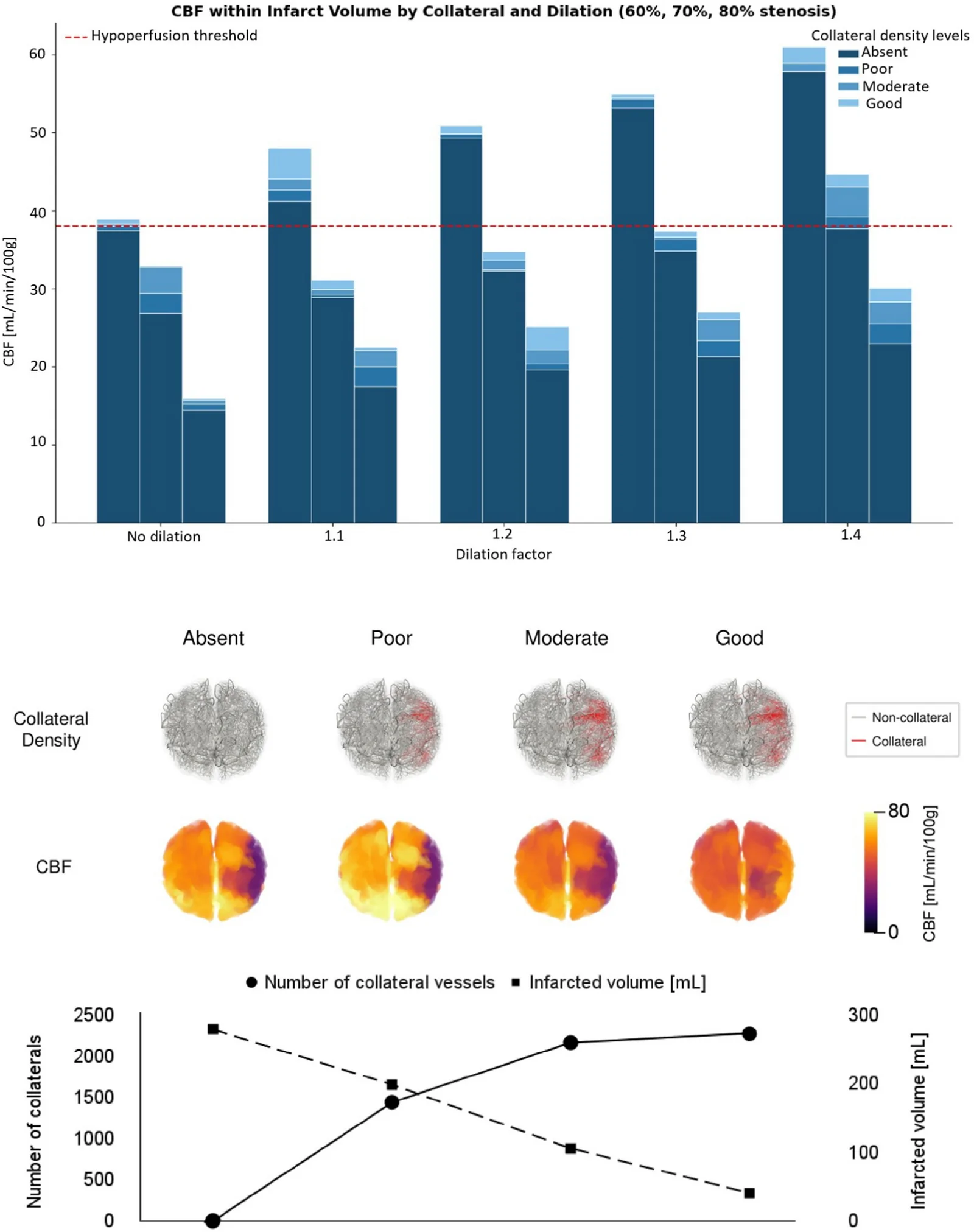
Top row: mean cerebral blood flow (mL/min/100 g) within the initial infarct volume for four collateral density levels and five vasodilation levels under 60 %, 70 %, and 80 % stenosis conditions. Second row: mean cerebral blood flow (mL/min/100 g) within the initial infarct volume for four collateral density levels without arteriolar vasodilation under 70 % stenosis. Collateral vessel configurations (red) for four density levels (absent, poor, moderate, and good) with corresponding tissue perfusion maps. Third row: number of generated collateral vessels and the resulting infarct tissue volumes.

The contributions of collateral circulation and arteriolar vasodilation to cerebral perfusion were evaluated using a subject-specific cerebrovascular model. A synthetic vascular network consisting of 32,320 segments was generated based on segmented cerebrovascular arteries. To establish a baseline, the initial infarct volume was identified as the region where perfusion fell below 60% of the metabolic demand under the condition of no vasodilation and absent collaterals. Three degrees of stenosis (60%, 70%, and 80% diameter narrowing) were introduced in the left middle cerebral artery (LMCA). For each stenosis level, all 20 combinations of collateral density and vasodilation were simulated, and perfusion distributions were analyzed. Perfusion simulations were conducted for 20 configurations, combining five levels of vasodilation capacity (0%, 10%, 20%, 30%, and 40%) with four collateral density levels: absent, poor, moderate, and good. Results for all collateral density levels are presented in Fig. 8. The middle and bottom rows correspond to the 70% stenosis case, representing the intermediate disease severity in our study. As collateral density increases, the infarcted volume decreases; however, even under the most favorable collateral configuration, a residual infarct persists. This finding indicates that, for moderate stenosis, collaterals alone are insufficient to fully restore perfusion above the ischemic threshold. Perfusion improvements were observed with increasing collateral density and arteriolar vasodilation capacity. For the 60% stenosis case, perfusion exceeded the hypoperfusion threshold for all collateral density levels with vasodilation above 10 %. However, as stenosis severity increased, fewer collateral configurations met the threshold, and no combination restored adequate perfusion for the 80% stenosis condition.

**Table 1:** Cerebral blood flow (CBF) within the infarcted region for collateral-only (poor, moderate, and good) and vasodilation-only (10 %, 20 %, 30 %, and 40 %) cases under 60 %, 70 %, and 80 % stenosis conditions

CBF (ml/min/100 g)	Case	60 % stenosis	70 % stenosis	80 % stenosis
collateral only	Poor	38.00	26.06	15.24
	Moderate	38.29	26.27	15.77
	Good	38.90	26.49	15.97
	10 %	44.73	28.91	17.38
Vasodilation only	20 %	49.33	32.23	19.62
	30 %	53.18	34.84	21.28
	40 %	57.82	37.71	22.92

Table 1 illustrates perfusion outcomes across different stenosis severities with collateral network or arteriolar vasodilation only. For the 60% stenosis case, the average perfusion within the initially infarcted region ranged between 38.00 ml/min/100 g and 57.82 ml/min/100 g, relative to the hypoperfusion threshold of 39 ml/min/100 g. For the 70% stenosis case, perfusion ranged from 26.06 to 37.71 ml/min/100 g. Under the 80% stenosis condition, the average perfusion values fell below the threshold across all configurations, indicating insufficient reperfusion. Across all stenosis severities, arteriolar vasodilation contributed more significantly to perfusion recovery than collateral density.

### Computation Time of the Idealized and Subject-Specific Models

Computation times for the major steps of the pipeline are summarized in Table 2 for both the idealized and subject-specific models. Segmentation is not required for the idealized case but constitutes a non-negligible component in the subject-specific workflow. Across both models, synthetic vessel generation and collateral network generation are the dominant computational steps. In particular, collateral generation represents a major computational bottleneck in the subject-specific case due to its current serial implementation, whereas perfusion simulations are comparatively inexpensive. All reported timings correspond to single-CPU execution.

**Table 2:** Computation time for each step of the pipeline for the idealized spherical and subject-specific cerebrovascular models. All timings correspond to single-CPU execution

Model	Segmentation	Synthetic vessel generation	Collateral generation	Perfusion simulation
Ideal	N/A	4 min	3 min	18 s
Cerebrovascular	5 h	7 h	8 h	33 min

## Discussion

We developed computational methods to quantify the relative contributions of collateral circulation and arteriolar vasodilation to tissue perfusion. An idealized vascular geometry extending from large arteries to the pre-capillary level was used to systematically investigate the effects of collateral density and radius ratio (see 3.1). The simulation results showed that collateral vessel radius exerts a dominant influence on perfusion. Specifically, when collateral vessels approach the size of primary arteries, they substantially augment perfusion by providing effective auxiliary pathways. In contrast, when collateral radii are small, increased network density improves perfusion but with a limited impact compared to that of vessel size. These findings suggest that collateral radius is a critical determinant in the efficacy of collateral circulation in restoring tissue perfusion. In comparison, for the stenosis degrees studied here, arteriolar vasodilation demonstrated a more effective role in improving perfusion, with collateral playing a secondary role under most conditions. However, it has been shown that larger collaterals like the circle of Willis have high impact on reperfusion. These larger collaterals are not included in the scope of this study.

The analysis further quantified the energetic costs associated with collateral circulation. The metabolic energy expenditure required to develop and maintain collateral vessels, along with the viscous energy loss associated with blood flow through these vessels, was quantified. As expected, metabolic energy expenditure increases with both the size and number of collaterals, reflecting the physiological cost of constructing and sustaining an extensive collateral network. In contrast, viscous energy loss does not exhibit a monotonic relationship with respect to collateral radius. When collateral vessels are small, viscous work increases with radius due to a substantial rise in flow rate, despite a reduction in pressure gradient. In this regime, the contribution to perfusion remains limited, and the network incurs a relatively high energetic cost to maintain even modest flow rates. However, when collateral vessels are moderately sized, viscous work decreases with increasing radius. This behavior is attributed to a sharp decline in pressure drop, which offsets the increase in flow, thereby reducing overall energy expenditure. This suggests that collaterals of moderate size may represent an energetically favorable configuration for restoring perfusion. As collateral radius continues to increase—approaching values large enough to be detected in imaging—the contribution to perfusion becomes maximal. In this range, viscous work becomes dominated by flow rate, as the pressure loss is minimal. A visualization of the pressure difference and flow within collateral can be seen Fig. 5. These results highlight the trade-off between metabolic cost and viscous energy loss, suggesting that the energetically optimal collateral configuration depends on both vessel size and density, and is likely subject-specific, influenced by disease severity and pathophysiology.

To evaluate variability, stochastic collateral networks with poor, moderate, and good densities were generated and calibrated against perfusion outcomes reported by Boer et al. [47]. The influence of stochastic properties decreased with increasing density. Under poor collateralization, variations in collateral distribution and radius had a strong impact on perfusion, whereas under moderate and good collateralization, perfusion became more consistent as the relative contribution of individual collaterals diminished.

For the subject-specific cerebrovascular model 3.3, simulations were conducted across varying degrees of arterial stenosis and collateral configurations to evaluate the combined effects of collateral networks and vasodilation on cerebral perfusion. The results further demonstrated that arteriolar vasodilation plays a dominant role in restoring perfusion under mild-to-moderate stenosis, consistent with the idealized model findings. However, as the severity of stenosis increased, the contribution of collateral circulation became increasingly significant. In the case of 90% diameter stenosis, vasodilation alone was insufficient to restore perfusion above the ischemic threshold, and collateral vessels were essential to augment blood flow to affected regions. Previous studies have demonstrated that although collateral circulation plays a critical role in preserving tissue viability under stenotic conditions in the cardiovascular system, it is often insufficient to fully restore perfusion to normal levels [9, 18, 24, 43, 45], which aligns with our findings.

Although the present study focuses on mechanistic insights using idealized and subject-specific models, the proposed framework can be further extended toward clinical translation by incorporating indicators of collateral and vasodilatory capacity. Direct imaging of arterioles and most collateral vessels remains beyond current clinical resolution, yet several indirect measurements are routinely used to estimate these compensatory capacities. For example, arterial spin labeling provides tissue perfusion maps that reflect collateral recruitment in the brain, while acetazolamide quantifies cerebrovascular dilatory capacity. Similarly, in coronary arteries, wedge pressure measurements or adenosine-induced hyperemia are commonly used to assess collateral flow and vasodilatory capacity. Integrating these surrogate measurements as boundary conditions or parameter constraints within our multiscale model would enable patient-specific estimation of compensatory mechanisms and improve prediction of perfusion deficits. This approach may ultimately support individualized risk assessment and treatment planning, especially in cases where imaging data on the microvasculature are limited.

### Limitations

Several limitations of this study must be acknowledged. First, the collateral network densities for both the idealized and subject-specific models were calibrated using studies that did not account for vessel dilation, which may lead to an overestimation of collateral density. Second, we modeled only arteriolar vasodilation, while other autoregulatory mechanisms—such as passive and myogenic responses—were not included. Because the present work focuses on steady blood flow and relatively small pressure variations, passive pressure-dependent responses were neglected. Moreover, the interaction between arteriolar vasodilation and myogenic regulation remains incompletely understood, and our assumption of an inhibited myogenic response to satisfy metabolic demand warrants further investigation. Third, all collateral vessels in the cerebrovascular model were assumed to be below imaging resolution, constraining their diameters to less than 1.0 mm. Additionally, the patient-specific simulations were limited to a single stenosis in the left middle cerebral artery, reducing the potential contribution of Circle of Willis pathways and other large-scale collaterals. In vascular configurations with strong macro-collateral pathways—such as an intact Circle of Willis or collaterals visible in clinical imaging—collateral recruitment may become the dominant compensatory mechanism for reperfusion and could exceed the contribution of arteriolar vasodilation. Fourth, the current model represents only the intraparenchymal arterial and arteriolar vasculature and does not include pial arterial networks, pial–pial collaterals, or collaterals involving branches of the external carotid circulation, which form a separate vascular domain [15]. Fifth, collateral segments were modeled as straight connections, which does not reflect their tortuosity in vivo. This simplification may underestimate the pressure drop across collateral vessels. Sixth, blood supply was assumed to occur primarily via advection through the vascular network, and diffusion-driven transport within the brain tissue was neglected. These assumptions may result in an underestimation of the full contribution of collateral circulation. Seventh, except for the density analysis in the idealized geometry, only one realization was simulated per configuration, preventing full characterization of stochastic variability. The density analysis in the idealized model also relied on a relatively small sample size due to computational cost. Finally, the computational cost of generating dense collateral networks remains a limitation of the present framework. Although the synthetic arterial tree algorithm is parallelized, collateral generation is currently performed in serial to preserve prescribed density distributions. Emerging rapid-prototyping methods for synthetic vasculature, such as the approach of Sexton et al. [44], together with further parallelization, could substantially reduce these costs and will be incorporated in future extensions of the pipeline.

### Concluding Remarks

We developed a multiscale computational framework to quantify the contributions of collateral circulation and arteriolar vasodilation to tissue perfusion under pathological conditions. Using both an idealized spherical geometry and a subject-specific cerebrovascular model, we systematically evaluated the impact of varying collateral density, vessel radius, and vasodilation capacity on perfusion outcomes. The results demonstrate that while vasodilation plays a dominant role in restoring perfusion under mild-to-moderate stenosis, the relative importance of collateral circulation increases with stenosis severity. Notably, perfusion recovery was found to be highly sensitive to collateral vessel radius, with smaller-radius networks showing limited efficacy despite increased density. Energetic analyses further revealed trade-offs between metabolic cost and viscous energy loss across different collateral configurations, suggesting an optimal range for collateral development depending on the disease severity and pathophysiological condition of each patient. The proposed computational framework offers a valuable tool for patient-specific assessment of ischemic risk and has the potential to support diagnostic and therapeutic decision-making in cardiovascular diseases.

## Data Availability

Data supporting the results of this study are available from the corresponding author upon reasonable request.

## Materials Availability

Materials supporting the results of this study are available from the corresponding author upon reasonable request.

## Code availability

Source code developed in this study is available from the corresponding author upon reasonable request.
